# Synthesis, crystal structure and Hirshfeld surface analysis of bis­[*N*-(4-chloro­benz­yl)-*N*-do­decyl­dithio­carbamato-κ^2^*S*,*S*′]palladium(II)

**DOI:** 10.1107/S205698902600188X

**Published:** 2026-03-24

**Authors:** R. Pallavi, N. Srinivasan, S. Thirumaran

**Affiliations:** a Department of Chemistry Annamalai University, Annamalainagar-608002 India; b Department of Chemistry Saveetha School of Engineering Saveetha Institute of Medical And Technical Sciences (SIMATS) Saveetha Nagar Thandalam Chennai 602105 Tamil Nadu India; Universidade de Sâo Paulo, Brazil

**Keywords:** crystal structure, Hirshfeld surface analysis, di­thio­carbamate, palladium

## Abstract

The title compound, [Pd(C_20_H_31_ClNS_2_)_2_], crystallizes about an inversion centre in the monoclinic space group *C*2/*c*. The Pd^II^ cation adopts a square-planar coordination geometry defined by four sulfur atoms from two *N*-(4-chloro­benz­yl)-*N*-do­decyl­dithio­carbamate anions. The crystal packing features C—H⋯S hydrogen bonds, which form a belt motif. Hirshfeld surface analysis indicates that H⋯H (62.9%), S⋯H/H⋯S (14.0%) and Cl⋯H/H⋯Cl (11.1%) contacts dominate the inter­molecular inter­actions.

## Abstract 2

The title compound crystallizes about an inversion centre in the monoclinic space group *C*2/*c*. The Pd^II^ cation adopts a square-planar coordination geometry defined by four sulfur atoms from two *N*-(4-chloro­benz­yl)-*N*-do­decyl­dithio­carbamate anions. The crystal packing features C—H⋯S hydrogen bonds, which form a belt motif.

## 1. Chemical context

Di­thio­carbamates (*R*_2_NCS_2_^−^) form stable complexes with transition metals, lanthanides and actinides (Hogarth *et al.*, 2005 ▸; Hitchcock *et al.*, 2004 ▸; Mahato *et al.*, 2015 ▸; Behrle *et al.*, 2018 ▸). They can bind to metals in nine distinct coordination modes. The most common of these are monodentate and chelating bidentate coordination modes (Hogarth *et al.*, 2005 ▸). The properties of complexes are influenced by the electronic configuration of the central metal cation and N-bonded organic (*R*) moiety of di­thio­carbamate ligands (Hogarth *et al.*, 2012 ▸; Godoy-Alcántar *et al.*, 2025 ▸). Metal–di­thio­carbamate complexes exhibit a wide range of applications in various fields, including catalysis, sensors, medicine, material science and rubber manufacturing (Ajiboye *et al.*, 2022 ▸). In particular, Pd^II^ di­thio­carbamate complexes show important biological activities, *viz*. anti­bacterial (Khan *et al.*, 2016 ▸), anti­fungal (Ferreira *et al.*, 2014 ▸), anti­cancer (Khan *et al.*, 2016 ▸; Khan *et al.*, 2011 ▸). Several studies on metal di­thio­carbamate complexes indicated that the length of the alkyl chain (*R*) of the di­thio­carbamate ligand enhances the solubility and activity of complexes (Hogarth *et al.*, 2012 ▸).
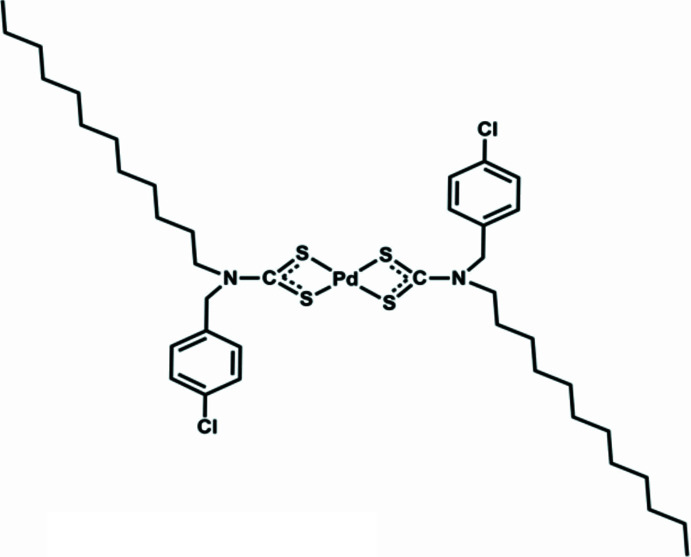


**Figure 1:**
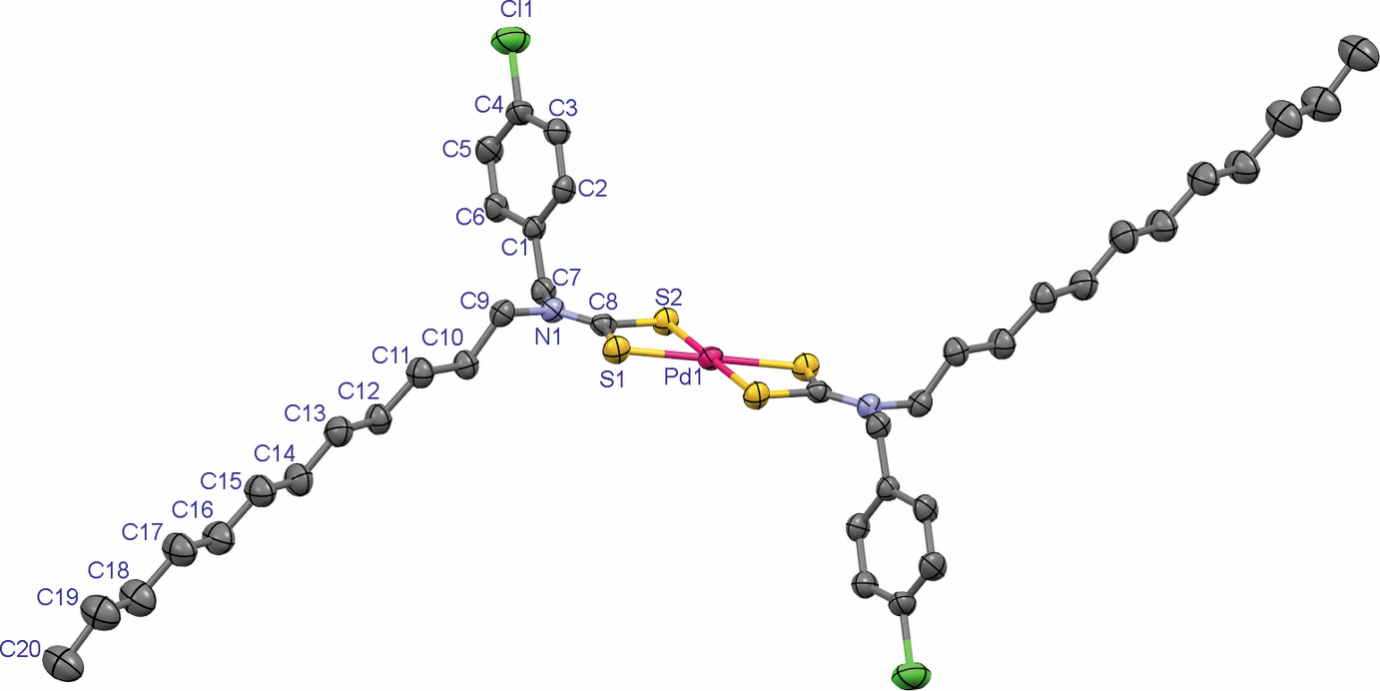
The mol­ecular structure of the title complex, showing the atom-labelling scheme. Displacement ellipsoids are drawn at the 30% probability level. Hydrogen atoms have been omitted for clarity. Symmetry-generated atoms were generated by the operation *x* + 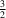, −*y* + 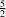, −*z* + 1.

As part of our work in this area, we report here the synthesis, crystal structure and Hirshfeld surface analysis of a Pd^II^ di­thio­carbamate complex containing a long-chain alkyl group (dodec­yl) (Fig. 1 ▸).

## 2. Structural commentary

The title complex crystallizes in the monoclinic space group *C2/c*. It is a four-coordinated structure in which the palladium atom is coordinated by four sulfur atoms from two *N*-(4-chloro­benz­yl)-*N*-dodecyldi­thio­carbamate ligands (Fig. 1 ▸). This four-coordinate geometry results in square planar spatial configuration. The bond parameters for the metal–ligand inter­actions are Pd—S1 = 2.3166 (12) Å, Pd—S2 = 2.3271 (10) Å and the chelate angle S1—Pd—S2 is 75.32 (4) °. These values are typical for Pd^II^ di­thio­carbamate complexes and indicate an even bonding of the palladium with the two ligand S atoms, without pronounced asymmetry in the bond lengths. For the metal coordination sphere, the calculated root-mean-square deviation = 0.00 Å, reflecting an almost perfect planarity of the S1, S2, S1^i^, S2^i^ coordination [symmetry code: (i) *x* + 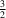, −*y* + 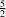, −*z* + 1] around the Pd^II^ atom. The τ_4_ index (Yang *et al.*, 2007 ▸) relative to the ideal square-planar geometry (*D*_4*h*_) was calculated to qu­anti­tatively assess the degree of distortion of four-coordinate environment around Pd^II^. For the title complex, the calculated τ_4_ value is 0.0, which suggests that the title complex has an ideal square-planar geometry.

The C8—N1 bond is significantly shorter than C7—N1 and C9—N1 bonds, an observation nsistent with a significant contribution of the *R*_2_NCS_2_^1−^ canonical form to the overall electronic structure of the di­thio­carbamate ligand. The C8—S1 and C8—S2 bond lengths are almost equal [Δ(C—S) = 0.008 Å], showing the symmetric bidentate coordination of di­thio­carbamate ligands.

## 3. Supra­molecular features and Hirshfeld surface analysis

**Figure 2:**
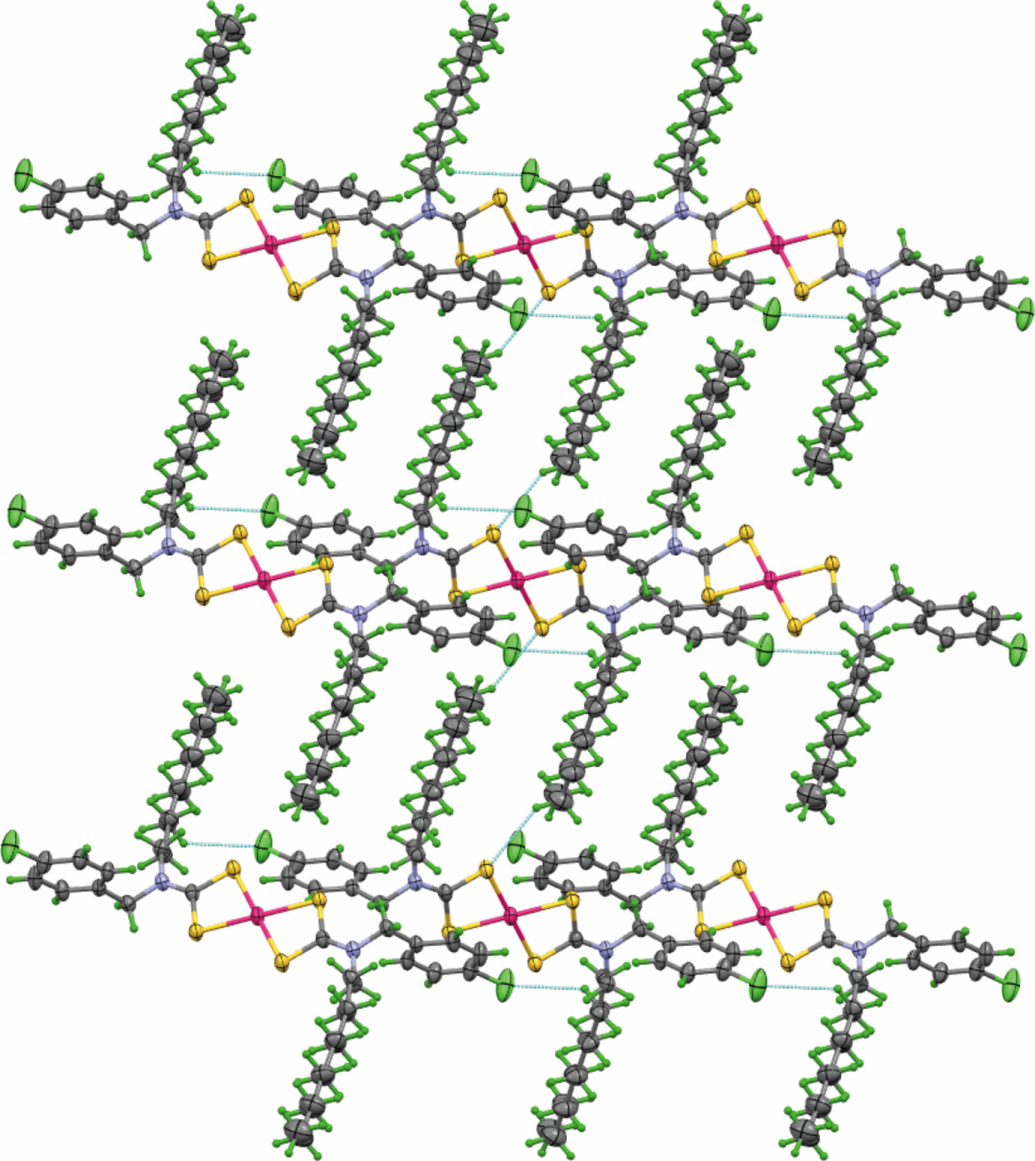
Packing of the title complex viewed along the *b* axis.

In the crystal, two C—H⋯S hydrogen bonds are observed between the sulfur atoms (S1 and S1^i^) of the di­thio­carbamate functional group and neighbouring methyl group hydrogen atoms (H19*B*), which leads to the formation of a belt motif along the *b*-axis direction. Additional C—H⋯Cl inter­actions further contribute to the supra­molecular arrangement (Table 1 ▸, Fig. 2 ▸).

**Figure 3:**
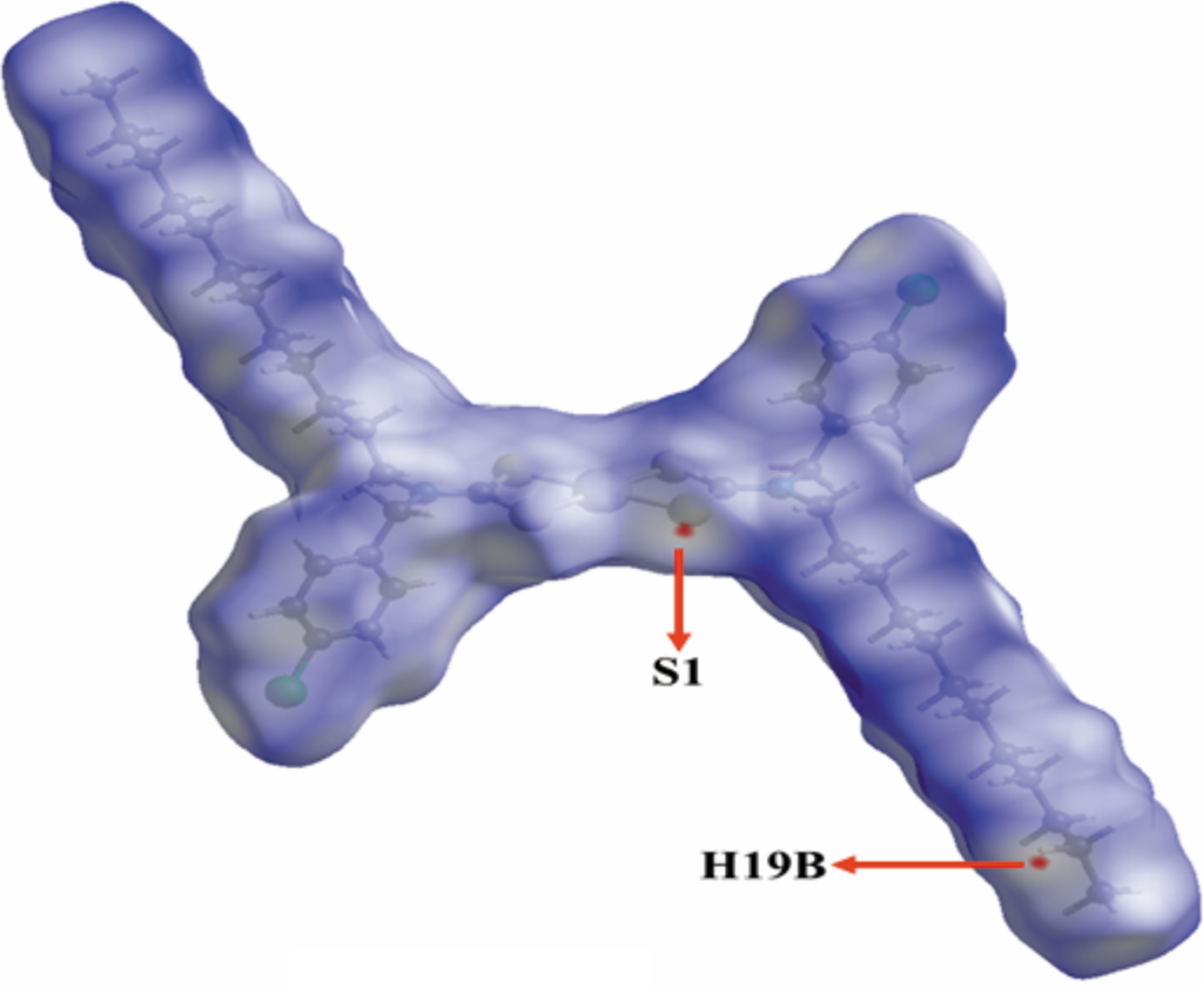
Hirshfeld surface mapped over *d*_norm_, showing close inter­molecular contacts, with red regions highlighting the S1⋯H19*B* inter­action.

A Hirshfeld surface analysis was performed using *CrystalExplorer 21.0* (Spackman *et al.*, 2021 ▸). The Hirshfeld surface and fingerprint plot calculations were performed on the entire mol­ecule. The Hirshfeld surface mapped with *d*_norm_ is shown in Fig. 3 ▸, where white regions indicate contacts at van der Waals separations, red spots denote shorter contacts (*e.g.* hydrogen bonds) and blue regions indicate longer contacts. Thus, the red regions of the surface correspond to the S1⋯H19*B* inter­action.

**Figure 4:**
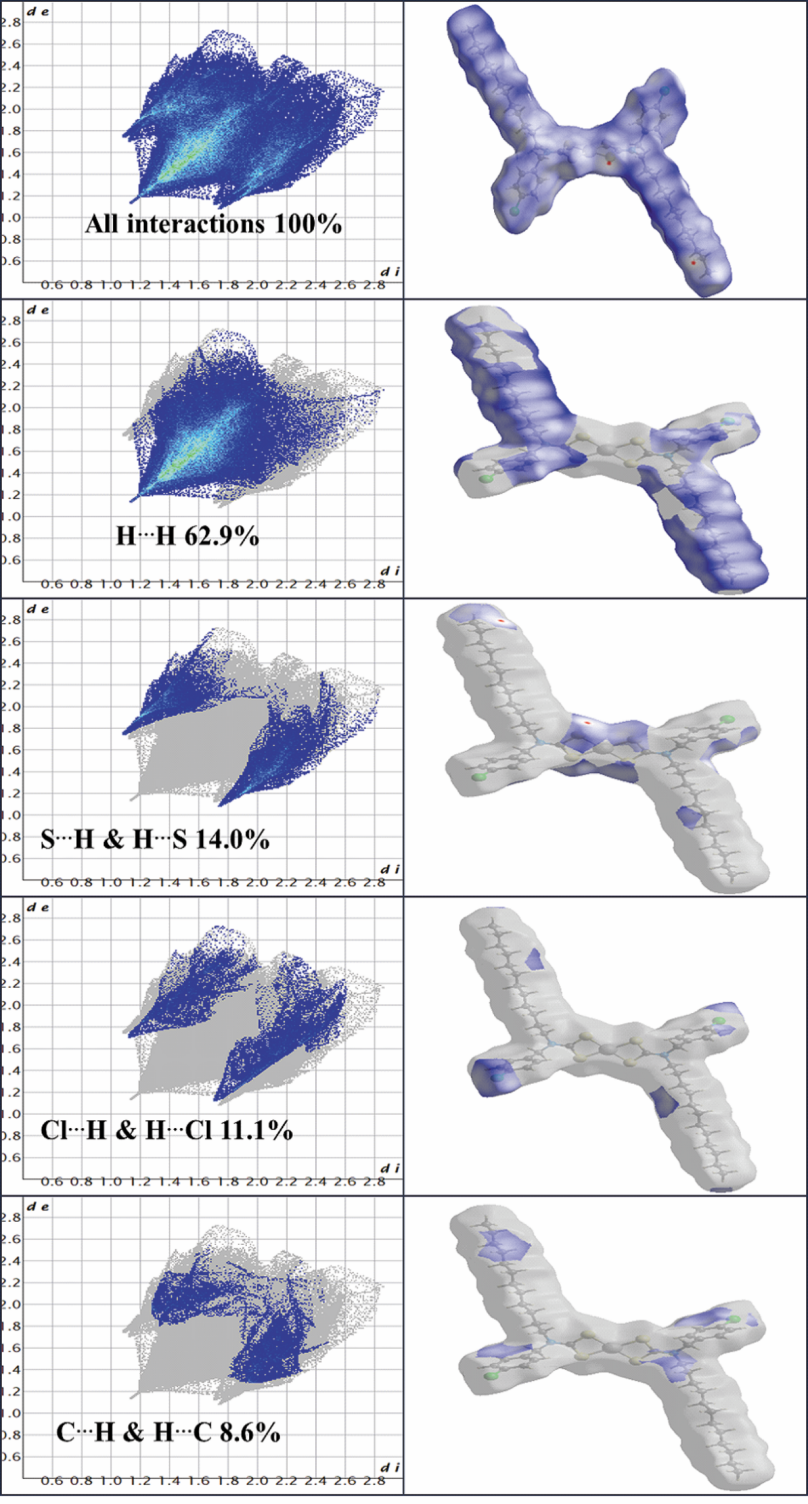
Two-dimensional fingerprint plots and the corresponding Hirshfeld surface mapped over *d*_norm_ for the title complex, showing the overall inter­molecular inter­actions and their relative contributions, including H⋯H (62.9%), S⋯H/H⋯S (14.0%), Cl⋯H/H⋯Cl (11.1%) and C⋯H/H⋯C (8.6%) contacts.

**Figure 5:**
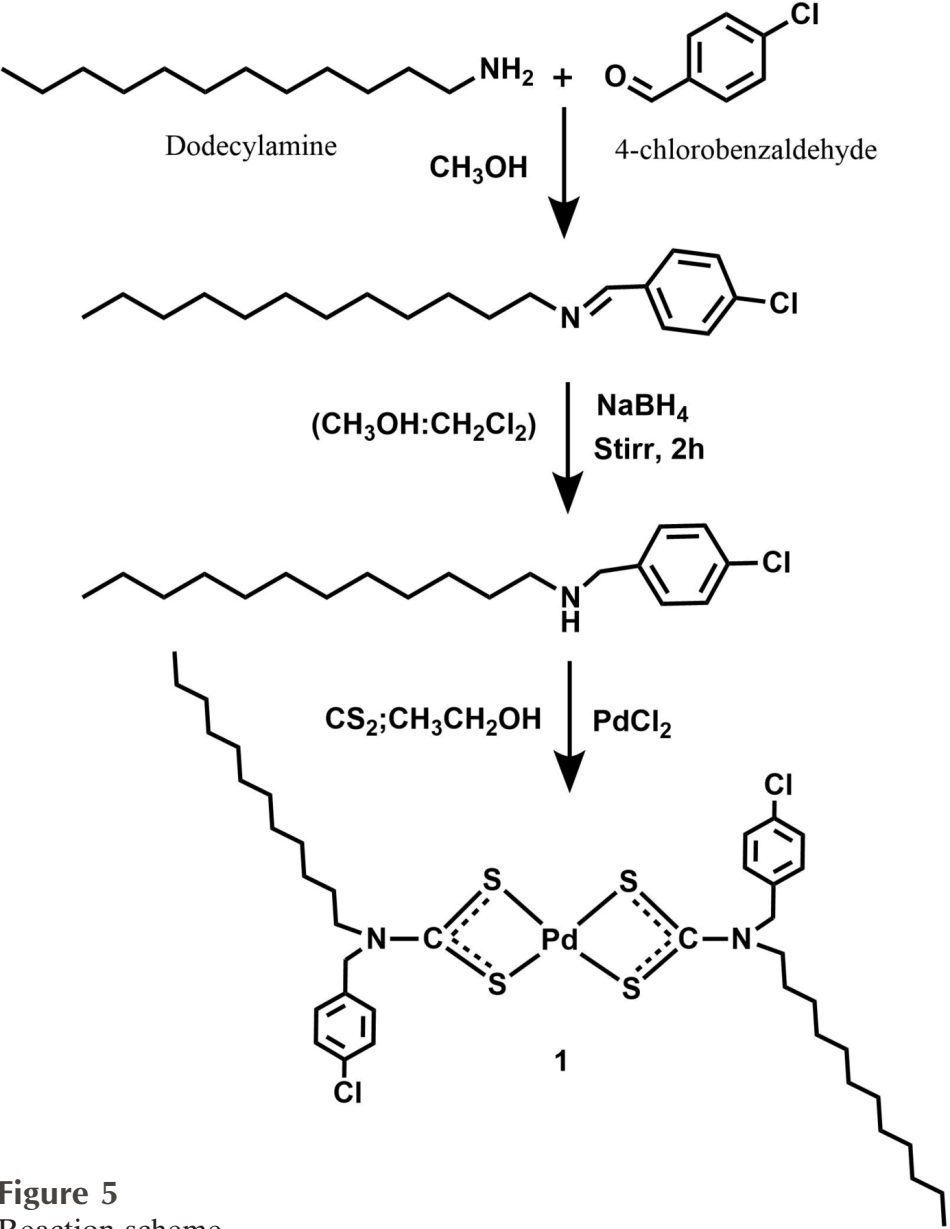
Reaction scheme.

The overall two-dimensional fingerprint plot (Fig. 4 ▸) shows that the largest contribution to the surface inter­actions arises from H⋯H contacts, accounting for 62.9%. This is typical for complexes with a high degree of hydrogen saturation and indicates dense mol­ecular packing (Tojiboyeva *et al.*, 2025 ▸). The S⋯H/H⋯S (14.0%) and Cl⋯H/H⋯Cl (11.1%) contacts are consistent with the crystal packing data (Fig. 5 ▸), while Pd⋯H/H⋯Pd, Pd⋯Cl/H⋯Cl, C⋯C, S⋯Cl/Cl⋯S, C⋯Cl/Cl⋯C and N⋯C/C⋯N make only a small contribution. The crystal-packing data reveal the presence of significant hydrogen-bonding inter­actions. Minor contributions (1%) from Pd⋯H/H⋯Pd are important because complexes with *M*⋯H inter­actions are believed to act as catalysts in the synthesis of organic compounds (Wang *et al.*, 2025 ▸)

## 4. Synthesis and crystallization

*N*-(4-chloro­benz­yl)-*N*-do­decyl­amine was synthesized (Fig. 5 ▸) by a procedure reported earlier (Gokul *et al.* 2025 ▸). 4-Chloro­benzaldehyde (5.3 mmol) and do­decyl­amine (4.6 mmol) were dissolved in methanol and the reaction mixture was stirred at room temperature for 4 h. After completion of the imine formation, sodium borohydride (13.6 mmol) was added portionwise to the reaction mixture under stirring. The reaction was allowed to proceed at room temperature until complete reduction was achieved. The solvent was removed and the amine was partitioned between di­chloro­methane and water. The organic layer was separated and evaporated to afford the corresponding amine. This was dissolved in 20 mL of ethanol. 4 mmol of NaOH in an aqueous solution were added to the amine solution followed by CS_2_ (4 mmol) at 278 K and stirred for 30 min. An aqueous solution of PdCl_2_ (2 mmol) was added to the reaction mixture. The obtained solid was purified by washing with ethanol and water and dried in a desiccator (yield: 61%). Single crystals appropriate for X-ray crystallographic analysis were successfully obtained by slow evaporation of a chloro­form–aceto­nitrile solution. M.p. 392–394 K. IR (ATR), *v* (cm^−1^): 1496 [C—N (thio­ureide)], 964 (C—S). ^1^H NMR (400MHz, CDCl_3_): δ 7.33 (*d*, *J* = 8.0 MHz, 4H), 7.24 (*d*, *J* = 8.4 MHz, 4H), 4.87 (*s*, 4H, 4-ClC_6_H_4_-C**H**_2_), 3.56 [*t*, *J* = 8.0 MHz, 4H, CH_3_-(CH_2_)_9_-CH_2_-C**H**_2_-N], 1.62 [*b*, 4H, (N-CH_2_-C**H**_2_-(CH_2_)_9_-CH_3_], 1.25 [*b*, 36H, CH_3_-(C**H**_2_)_9_-CH_2_-CH_2_-N], 0.88 {*t*, *J* = 7.2 MHz, 6H [C**H**_3_-(CH_2_)_9_-CH_2_-CH_2_-N]}. ^13^C{^1^H} NMR (100.6 MHz, CDCl_3_): δ 129.1, 129.5, 132.9, 134.2 (aromatic carbons), 22.7, 26.7, 26.8, 29.1, 29.3, 29.4, 29.5, 29.6, 31.9 [CH_3_-(**C**H_2_)_10_-CH_2_-N], 14.1 [**C**H_3_-(CH_2_)_10_-CH_2_-N], 51.5 (4-ClC_6_H_4_-**C**H_2_-N), 49.1 [CH_3_-(CH_2_)**C**H_2_-N]. Analysis calculated for C_40_H_62_Cl_2_N_2_PdS_4_: C; 54.81, H; 7.13, N; 3.20. Found: C; 54.61, H; 7.09, N; 3.18.

## 5. Refinement

Crystal data, data collection and structure refinement details are summarized in Table 2 ▸. H atoms were positioned geometrically (C—H = 0.93–0.97 Å) and refined as riding with *U*_iso_(H) = 1.2–1.5*U*_eq_(C).

## Supplementary Material

Crystal structure: contains datablock(s) I. DOI: 10.1107/S205698902600188X/ex2098sup1.cif

Structure factors: contains datablock(s) I. DOI: 10.1107/S205698902600188X/ex2098Isup2.hkl

CCDC reference: 2532016

Additional supporting information:  crystallographic information; 3D view; checkCIF report

## Figures and Tables

**Table 1:** Hydrogen-bond geometry (Å, °) *Cg*1 is the centroid of the C1–C6 ring.

*D*—H⋯*A*	*D*—H	H⋯*A*	*D*⋯*A*	*D*—H⋯*A*
C19—H19*B*⋯S1^i^	0.97	2.91	3.676 (6)	137
C12—H12*A*⋯Cl1^ii^	0.97	2.90	3.602 (2)	130
C10—H10*B*⋯*Cg*1^iii^	0.97	3.24	3.982 (3)	134

Symmetry codes: (i) 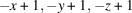; (ii) 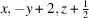; (iii) 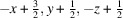.

**Table 2:** Experimental details

Crystal data	
Chemical formula	[Pd(C_20_H_31_ClNS_2_)_2_]
*M*_r_	876.46
Crystal system, space group	Monoclinic, *C*2/*c*
Temperature (K)	300
*a*, *b*, *c* (Å)	24.348 (2), 10.0966 (10), 18.3551 (17)
β (°)	91.168 (3)
*V* (Å^3^)	4511.3 (7)
*Z*	4
Radiation type	Mo *K*α
μ (mm^−1^)	0.74
Crystal size (mm)	0.14 × 0.12 × 0.05

Data collection	
Diffractometer	Bruker D8 QUEST
Absorption correction	Multi-scan (*SADABS*; Krause *et al.*, 2015 ▸)
*T*_min_, *T*_max_	0.904, 0.965
No. of measured, independent and observed [*I* > 2σ(*I*)] reflections	52590, 5647, 2891
*R*_int_	0.089

Refinement	
*R*[*F*^2^ > 2σ(*F*^2^)], *wR*(*F*^2^), *S*	0.061, 0.110, 1.14
No. of reflections	5647
No. of parameters	224
H-atom treatment	H-atom parameters constrained
Δρ_max_, Δρ_min_ (e Å^−3^)	0.44, −0.42

Computer programs: *APEX2* and *SAINT* (Bruker, 2007 ▸), *SHELXT2018/2* (Sheldrick, 2015*a* ▸), *SHELXL2019/2* (Sheldrick, 2015*b* ▸) and *ShelXle* (Hübschle *et al.*, 2011 ▸).

## supplementary crystallographic information

### Bis[N-(4-chlorobenzyl)-N-dodecyldithiocarbamato-κ2S,S']palladium(II) Crystal data

**Table:**

[Pd(C_20_H_31_ClNS_2_)_2_]	*F*(000) = 1840
*M**_r_* = 876.46	*D*_x_ = 1.291 Mg m^−^^3^
Monoclinic, *C*2/*c*	Mo *K*α radiation, λ = 0.71073 Å
*a* = 24.348 (2) Å	Cell parameters from 9549 reflections
*b* = 10.0966 (10) Å	θ = 2.2–24.0°
*c* = 18.3551 (17) Å	µ = 0.74 mm^−^^1^
β = 91.168 (3)°	*T* = 300 K
*V* = 4511.3 (7) Å^3^	Block, gold
*Z* = 4	0.14 × 0.12 × 0.05 mm

### Bis[N-(4-chlorobenzyl)-N-dodecyldithiocarbamato-κ2S,S']palladium(II) Data collection

**Table:**

Bruker D8 QUEST diffractometer	5647 independent reflections
Radiation source: microfocus sealed tube, INCOATEC IµS 3.0	2891 reflections with *I* > 2σ(*I*)
Multilayer mirror monochromator	*R*_int_ = 0.089
Detector resolution: 7.391 pixels mm^-1^	θ_max_ = 28.4°, θ_min_ = 2.2°
ω and φ scans	*h* = −32→32
Absorption correction: multi-scan (SADABS; Krause *et al.*, 2015)	*k* = −13→13
*T*_min_ = 0.904, *T*_max_ = 0.965	*l* = −24→24
52590 measured reflections	

### Bis[N-(4-chlorobenzyl)-N-dodecyldithiocarbamato-κ2S,S']palladium(II) Refinement

**Table:**

Refinement on *F*^2^	0 restraints
Least-squares matrix: full	Hydrogen site location: inferred from neighbouring sites
*R*[*F*^2^ > 2σ(*F*^2^)] = 0.061	H-atom parameters constrained
*wR*(*F*^2^) = 0.110	*w* = 1/[σ^2^(*F*_o_^2^) + (0.0142*P*)^2^ + 9.2012*P*]
*S* = 1.14	(Δ/σ)_max_ < 0.001
5647 reflections	Δρ_max_ = 0.44 e Å^−^^3^
224 parameters	Δρ_min_ = −0.42 e Å^−^^3^

### Bis[N-(4-chlorobenzyl)-N-dodecyldithiocarbamato-κ2S,S']palladium(II) Fractional atomic coordinates and isotropic or equivalent isotropic displacement parameters (Å^2^)

**Table:**

	*x*	*y*	*z*	*U*_iso_*/*U*_eq_	
Pd1	0.750000	1.250000	0.500000	0.06117 (16)	
Cl1	0.64664 (9)	1.42872 (16)	0.00819 (8)	0.1468 (7)	
S1	0.68053 (5)	1.11956 (12)	0.45024 (5)	0.0715 (3)	
S2	0.77378 (5)	1.22145 (11)	0.37885 (5)	0.0687 (3)	
N1	0.69935 (13)	1.0645 (3)	0.30989 (16)	0.0595 (9)	
C1	0.70804 (16)	1.1576 (4)	0.1853 (2)	0.0574 (10)	
C2	0.68411 (18)	1.2774 (4)	0.2019 (2)	0.0688 (12)	
H2	0.680701	1.302004	0.250375	0.083*	
C3	0.66525 (19)	1.3609 (4)	0.1480 (2)	0.0761 (13)	
H3	0.649283	1.441608	0.159793	0.091*	
C4	0.6702 (2)	1.3242 (5)	0.0765 (2)	0.0832 (14)	
C5	0.6933 (2)	1.2056 (5)	0.0588 (2)	0.0880 (15)	
H5	0.696394	1.181219	0.010160	0.106*	
C6	0.71187 (19)	1.1229 (4)	0.1130 (2)	0.0736 (13)	
H6	0.727339	1.041857	0.100787	0.088*	
C7	0.73172 (16)	1.0684 (4)	0.2435 (2)	0.0641 (11)	
H7A	0.734460	0.979389	0.224102	0.077*	
H7B	0.768595	1.098199	0.256033	0.077*	
C8	0.71556 (16)	1.1252 (4)	0.3701 (2)	0.0578 (10)	
C9	0.64853 (17)	0.9868 (4)	0.3065 (2)	0.0677 (12)	
H9A	0.629255	1.005795	0.260961	0.081*	
H9B	0.625118	1.014413	0.345899	0.081*	
C10	0.65798 (17)	0.8399 (4)	0.3121 (2)	0.0695 (12)	
H10A	0.675688	0.809339	0.268330	0.083*	
H10B	0.682612	0.822043	0.353150	0.083*	
C11	0.60555 (18)	0.7636 (5)	0.3217 (2)	0.0798 (13)	
H11A	0.590424	0.786847	0.368486	0.096*	
H11B	0.579308	0.791221	0.284252	0.096*	
C12	0.61147 (18)	0.6160 (5)	0.3182 (2)	0.0779 (13)	
H12A	0.641449	0.589028	0.350463	0.093*	
H12B	0.620959	0.590964	0.268979	0.093*	
C13	0.55992 (19)	0.5430 (5)	0.3392 (3)	0.0909 (15)	
H13A	0.549204	0.573552	0.386946	0.109*	
H13B	0.530705	0.566729	0.304935	0.109*	
C14	0.5644 (2)	0.3959 (5)	0.3413 (3)	0.0973 (16)	
H14A	0.593812	0.371267	0.375025	0.117*	
H14B	0.574098	0.364244	0.293312	0.117*	
C15	0.5115 (2)	0.3280 (5)	0.3642 (3)	0.1018 (17)	
H15A	0.502421	0.357928	0.412689	0.122*	
H15B	0.481910	0.354883	0.331267	0.122*	
C16	0.5149 (2)	0.1805 (6)	0.3644 (3)	0.1073 (18)	
H16A	0.525073	0.151296	0.316190	0.129*	
H16B	0.544204	0.154156	0.398044	0.129*	
C17	0.4637 (2)	0.1101 (6)	0.3850 (3)	0.1143 (19)	
H17A	0.434238	0.135662	0.351527	0.137*	
H17B	0.453546	0.137875	0.433484	0.137*	
C18	0.4695 (2)	−0.0404 (6)	0.3840 (4)	0.123 (2)	
H18A	0.478499	−0.067410	0.334920	0.147*	
H18B	0.500277	−0.064610	0.415632	0.147*	
C19	0.4224 (2)	−0.1139 (6)	0.4060 (4)	0.128 (2)	
H19A	0.392454	−0.096021	0.371693	0.153*	
H19B	0.411336	−0.081435	0.453262	0.153*	
C20	0.4301 (3)	−0.2613 (6)	0.4111 (4)	0.141 (2)	
H20A	0.397226	−0.301396	0.428682	0.212*	
H20B	0.460211	−0.280590	0.444016	0.212*	
H20C	0.437974	−0.296058	0.363713	0.212*	

### Bis[N-(4-chlorobenzyl)-N-dodecyldithiocarbamato-κ2S,S']palladium(II) Atomic displacement parameters (Å^2^)

**Table:**

	*U*^11^	*U*^22^	*U*^33^	*U*^12^	*U*^13^	*U*^23^
Pd1	0.0802 (3)	0.0613 (3)	0.0421 (2)	−0.0022 (3)	0.0038 (2)	0.0030 (2)
Cl1	0.253 (2)	0.1099 (12)	0.0762 (10)	0.0300 (13)	−0.0338 (11)	0.0153 (8)
S1	0.0860 (8)	0.0804 (8)	0.0484 (6)	−0.0111 (6)	0.0111 (6)	0.0011 (6)
S2	0.0813 (8)	0.0776 (8)	0.0474 (6)	−0.0123 (6)	0.0082 (5)	0.0001 (5)
N1	0.070 (2)	0.062 (2)	0.046 (2)	0.0002 (18)	0.0076 (17)	−0.0028 (16)
C1	0.069 (3)	0.058 (3)	0.045 (2)	−0.006 (2)	0.003 (2)	−0.003 (2)
C2	0.091 (3)	0.070 (3)	0.045 (2)	0.002 (2)	−0.001 (2)	−0.016 (2)
C3	0.102 (4)	0.064 (3)	0.062 (3)	0.008 (3)	−0.004 (3)	−0.008 (2)
C4	0.124 (4)	0.067 (3)	0.058 (3)	−0.003 (3)	−0.012 (3)	0.007 (2)
C5	0.139 (5)	0.079 (4)	0.046 (3)	−0.004 (3)	0.002 (3)	−0.005 (2)
C6	0.105 (4)	0.066 (3)	0.050 (3)	−0.004 (3)	0.009 (2)	−0.012 (2)
C7	0.071 (3)	0.067 (3)	0.055 (3)	0.001 (2)	0.009 (2)	−0.007 (2)
C8	0.074 (3)	0.053 (2)	0.047 (2)	0.007 (2)	0.005 (2)	0.0039 (19)
C9	0.068 (3)	0.076 (3)	0.059 (3)	−0.001 (2)	−0.001 (2)	−0.004 (2)
C10	0.072 (3)	0.069 (3)	0.068 (3)	−0.004 (2)	0.009 (2)	−0.007 (2)
C11	0.077 (3)	0.084 (4)	0.079 (3)	−0.011 (3)	0.008 (2)	−0.001 (3)
C12	0.082 (3)	0.079 (4)	0.073 (3)	−0.015 (3)	0.008 (2)	−0.003 (3)
C13	0.083 (4)	0.093 (4)	0.096 (4)	−0.013 (3)	−0.008 (3)	0.008 (3)
C14	0.087 (4)	0.097 (4)	0.107 (4)	−0.019 (3)	0.000 (3)	0.004 (3)
C15	0.085 (4)	0.093 (4)	0.127 (5)	−0.014 (3)	−0.001 (3)	0.014 (4)
C16	0.082 (4)	0.104 (5)	0.136 (5)	−0.010 (3)	−0.003 (3)	0.009 (4)
C17	0.090 (4)	0.102 (5)	0.151 (5)	−0.018 (4)	0.006 (4)	0.022 (4)
C18	0.100 (5)	0.116 (5)	0.153 (6)	−0.004 (4)	0.022 (4)	0.009 (4)
C19	0.113 (5)	0.120 (5)	0.151 (6)	−0.004 (4)	0.032 (4)	0.023 (4)
C20	0.127 (5)	0.109 (5)	0.189 (7)	0.003 (4)	0.025 (5)	0.020 (5)

### Bis[N-(4-chlorobenzyl)-N-dodecyldithiocarbamato-κ2S,S']palladium(II) Geometric parameters (Å, º)

**Table:**

Pd1—S1^i^	2.3165 (12)	C11—H11A	0.9700
Pd1—S1	2.3166 (12)	C11—H11B	0.9700
Pd1—S2^i^	2.3271 (10)	C12—C13	1.513 (6)
Pd1—S2	2.3271 (10)	C12—H12A	0.9700
Cl1—C4	1.728 (5)	C12—H12B	0.9700
S1—C8	1.716 (4)	C13—C14	1.489 (6)
S2—C8	1.724 (4)	C13—H13A	0.9700
N1—C8	1.318 (4)	C13—H13B	0.9700
N1—C7	1.465 (4)	C14—C15	1.526 (6)
N1—C9	1.465 (5)	C14—H14A	0.9700
C1—C6	1.379 (5)	C14—H14B	0.9700
C1—C2	1.379 (5)	C15—C16	1.491 (6)
C1—C7	1.503 (5)	C15—H15A	0.9700
C2—C3	1.372 (5)	C15—H15B	0.9700
C2—H2	0.9300	C16—C17	1.491 (6)
C3—C4	1.371 (6)	C16—H16A	0.9700
C3—H3	0.9300	C16—H16B	0.9700
C4—C5	1.364 (6)	C17—C18	1.526 (7)
C5—C6	1.369 (6)	C17—H17A	0.9700
C5—H5	0.9300	C17—H17B	0.9700
C6—H6	0.9300	C18—C19	1.432 (7)
C7—H7A	0.9700	C18—H18A	0.9700
C7—H7B	0.9700	C18—H18B	0.9700
C9—C10	1.505 (5)	C19—C20	1.503 (7)
C9—H9A	0.9700	C19—H19A	0.9700
C9—H9B	0.9700	C19—H19B	0.9700
C10—C11	1.504 (5)	C20—H20A	0.9600
C10—H10A	0.9700	C20—H20B	0.9600
C10—H10B	0.9700	C20—H20C	0.9600
C11—C12	1.498 (6)		
			
S1^i^—Pd1—S1	180.0	H11A—C11—H11B	107.5
S1^i^—Pd1—S2^i^	75.32 (4)	C11—C12—C13	113.1 (4)
S1—Pd1—S2^i^	104.68 (4)	C11—C12—H12A	109.0
S1^i^—Pd1—S2	104.67 (4)	C13—C12—H12A	109.0
S1—Pd1—S2	75.33 (4)	C11—C12—H12B	109.0
S2^i^—Pd1—S2	180.0	C13—C12—H12B	109.0
C8—S1—Pd1	86.98 (14)	H12A—C12—H12B	107.8
C8—S2—Pd1	86.47 (13)	C14—C13—C12	115.6 (4)
C8—N1—C7	121.9 (3)	C14—C13—H13A	108.4
C8—N1—C9	121.5 (3)	C12—C13—H13A	108.4
C7—N1—C9	116.6 (3)	C14—C13—H13B	108.4
C6—C1—C2	118.2 (4)	C12—C13—H13B	108.4
C6—C1—C7	120.0 (4)	H13A—C13—H13B	107.4
C2—C1—C7	121.8 (3)	C13—C14—C15	113.2 (5)
C3—C2—C1	121.2 (4)	C13—C14—H14A	108.9
C3—C2—H2	119.4	C15—C14—H14A	108.9
C1—C2—H2	119.4	C13—C14—H14B	108.9
C4—C3—C2	119.3 (4)	C15—C14—H14B	108.9
C4—C3—H3	120.4	H14A—C14—H14B	107.8
C2—C3—H3	120.4	C16—C15—C14	113.7 (5)
C5—C4—C3	120.7 (4)	C16—C15—H15A	108.8
C5—C4—Cl1	119.7 (4)	C14—C15—H15A	108.8
C3—C4—Cl1	119.6 (4)	C16—C15—H15B	108.8
C4—C5—C6	119.6 (4)	C14—C15—H15B	108.8
C4—C5—H5	120.2	H15A—C15—H15B	107.7
C6—C5—H5	120.2	C17—C16—C15	115.4 (5)
C5—C6—C1	121.2 (4)	C17—C16—H16A	108.4
C5—C6—H6	119.4	C15—C16—H16A	108.4
C1—C6—H6	119.4	C17—C16—H16B	108.4
N1—C7—C1	113.7 (3)	C15—C16—H16B	108.4
N1—C7—H7A	108.8	H16A—C16—H16B	107.5
C1—C7—H7A	108.8	C16—C17—C18	113.2 (5)
N1—C7—H7B	108.8	C16—C17—H17A	108.9
C1—C7—H7B	108.8	C18—C17—H17A	108.9
H7A—C7—H7B	107.7	C16—C17—H17B	108.9
N1—C8—S1	123.9 (3)	C18—C17—H17B	108.9
N1—C8—S2	124.9 (3)	H17A—C17—H17B	107.8
S1—C8—S2	111.2 (2)	C19—C18—C17	115.9 (5)
N1—C9—C10	113.4 (3)	C19—C18—H18A	108.3
N1—C9—H9A	108.9	C17—C18—H18A	108.3
C10—C9—H9A	108.9	C19—C18—H18B	108.3
N1—C9—H9B	108.9	C17—C18—H18B	108.3
C10—C9—H9B	108.9	H18A—C18—H18B	107.4
H9A—C9—H9B	107.7	C18—C19—C20	115.4 (5)
C11—C10—C9	112.6 (4)	C18—C19—H19A	108.4
C11—C10—H10A	109.1	C20—C19—H19A	108.4
C9—C10—H10A	109.1	C18—C19—H19B	108.4
C11—C10—H10B	109.1	C20—C19—H19B	108.4
C9—C10—H10B	109.1	H19A—C19—H19B	107.5
H10A—C10—H10B	107.8	C19—C20—H20A	109.5
C12—C11—C10	114.9 (4)	C19—C20—H20B	109.5
C12—C11—H11A	108.5	H20A—C20—H20B	109.5
C10—C11—H11A	108.5	C19—C20—H20C	109.5
C12—C11—H11B	108.5	H20A—C20—H20C	109.5
C10—C11—H11B	108.5	H20B—C20—H20C	109.5
			
C6—C1—C2—C3	−0.9 (6)	C9—N1—C8—S2	−178.5 (3)
C7—C1—C2—C3	176.6 (4)	Pd1—S1—C8—N1	178.9 (3)
C1—C2—C3—C4	0.2 (7)	Pd1—S1—C8—S2	−1.72 (18)
C2—C3—C4—C5	0.4 (8)	Pd1—S2—C8—N1	−179.0 (3)
C2—C3—C4—Cl1	−179.9 (4)	Pd1—S2—C8—S1	1.72 (18)
C3—C4—C5—C6	−0.2 (8)	C8—N1—C9—C10	−101.2 (4)
Cl1—C4—C5—C6	−179.9 (4)	C7—N1—C9—C10	77.4 (4)
C4—C5—C6—C1	−0.5 (7)	N1—C9—C10—C11	170.0 (3)
C2—C1—C6—C5	1.0 (7)	C9—C10—C11—C12	172.7 (4)
C7—C1—C6—C5	−176.5 (4)	C10—C11—C12—C13	171.4 (4)
C8—N1—C7—C1	−105.6 (4)	C11—C12—C13—C14	−176.3 (4)
C9—N1—C7—C1	75.8 (4)	C12—C13—C14—C15	178.9 (4)
C6—C1—C7—N1	−144.0 (4)	C13—C14—C15—C16	178.4 (5)
C2—C1—C7—N1	38.6 (5)	C14—C15—C16—C17	−178.7 (5)
C7—N1—C8—S1	−177.8 (3)	C15—C16—C17—C18	179.7 (5)
C9—N1—C8—S1	0.7 (5)	C16—C17—C18—C19	177.6 (6)
C7—N1—C8—S2	2.9 (5)	C17—C18—C19—C20	−174.9 (6)

Symmetry code: (i) −*x*+3/2, −*y*+5/2, −*z*+1.

### Bis[N-(4-chlorobenzyl)-N-dodecyldithiocarbamato-κ2S,S']palladium(II) Hydrogen-bond geometry (Å, º)

*Cg*1 is the centroid of the C1–C6 ring.

**Table:**

*D*—H···*A*	*D*—H	H···*A*	*D*···*A*	*D*—H···*A*
C19—H19*B*···S1^ii^	0.97	2.91	3.676 (6)	137
C12—H12*A*···Cl1^iii^	0.97	2.90	3.602 (2)	130
C10—H10*B*···*Cg*1^iv^	0.97	3.24	3.982 (3)	134

Symmetry codes: (ii) −*x*+1, −*y*+1, −*z*+1; (iii) *x*, −*y*+2, *z*+1/2; (iv) −*x*+3/2, *y*+1/2, −*z*+1/2.
